# Prof. Henri Bouley

**Published:** 1886-01

**Authors:** 


### OBITUARY.

### PROF. HENRI BOULEY.

The sad news has reached us of the death in Paris of this eminent scientist, at the age of seventy-one.

At the time of his death he was President of the Academy of Sciences, Paris, the highest Scientific Society in Europe, and Inspector-General of the Veterinary Schools of France.

